# Thyroglossal duct cyst on the suprasternal region: An extremely unusual location

**DOI:** 10.1016/j.ijscr.2023.108752

**Published:** 2023-08-28

**Authors:** Dereje G. Andargie, Yonas T. Habtemariam, Tizazu Y. Ayele, Mulat A. Agegnehu, Melesse G. Biadiglign, Amanuel Sisay Endeshaw

**Affiliations:** a Department of Surgery, Bahir Dar University College of Medicine and Health science, Bahir Dar, Ethiopia; b Department of Ophthalmology, Bahir Dar University College of Medicine and Health science, Bahir Dar, Ethiopia; c Department of Anesthesia, Bahir Dar University College of Medicine and Health science, Bahir Dar, Ethiopia

**Keywords:** Thyroglossal duct cyst, Suprasternal thyroglossal duct cyst, Neck mass, Sistrunk procedure

## Abstract

**Introduction and importance:**

A thyroglossal duct cyst (TGDC) is the most common cause of congenital midline anterior neck mass. It arises as a cystic expansion of a remnant of the thyroglossal duct anywhere between the foramen cecum of tongue and the isthmus of the thyroid. They are found in juxtaposition to the hyoid bone in 85 % of cases. Based on the obtainable information, it has been documented that there exist only two reported instances of a thyroglossal duct cyst occurring on the suprasternal region, which is contemplated as an atypical location for TGDCs. The atypical location of the swelling and difficulty of diagnosis made us report this case.

**Case presentation:**

This case report describes a 30 years old female patient who presented with a suprasternal swelling which was diagnosed to be a suprasternal TGDC after histopathology and a review of the literature on this topic.

**Clinical discussion:**

Thyroglossal duct cysts are usually asymptomatic, but may occasionally be infected by bacteria in the oral cavity, prompting the patient to seek medical care. It is mainly diagnosed at an early age if it is located on common anatomic areas. Later age of presentation or an unusual site like in this case makes the diagnosis difficult. Sistrunk procedure is the treatment of choice.

**Conclusion:**

Thyroglossal duct cyst at the suprasternal location is a very rare occurrence, but should be considered by the evaluating surgeon when he/she encounters midline neck swellings across all age groups.

## Highlights

• A thyroglossal duct cyst (TGDC) is the most common midline congenital anterior neck mass and is found in juxtaposition to the hyoid bone in 85 % of cases.
• Thyroglossal duct cysts are seen in children under 6 years of age in 76% of cases.
• TGDCs are usually asymptomatic, but may occasionally be infected by oral bacteria.
• Even though thyroglossal duct cyst at the suprasternal area is a very rare occurrence it is still a possibility and it should be in the list of surgeon’s differentials diagnosis for a suprasternal mass.

## 1 Introduction

Thyroglossal duct cyst (TGDC) is the first in the list of common midline congenital neck masses, contributing for 70 % of congenital neck anomalies [1]. The thyroglossal duct cyst typically manifests as a painless swelling in the midline of the neck and is commonly encountered below the hyoid bone in 85 % of cases. However, these cysts can also be found anywhere along the path between the foramen caecum of tongue and the suprasternal notch [2]. Nonetheless the hyoid, thyrohyoid membrane, or thyroid cartilage are often very closely associated with the cyst [3]. Approximately 90 % of the patient population exhibits symptoms before age 10, while a secondary cohort of patients manifests symptoms during the early stages of adulthood. There have been no reports of gender predilection [1]. The cyst classically moves upward on protruding the tongue, which is virtually pathognomonic of it [2]. Based on the obtainable information, it has been documented that there exist only two reported instances of a thyroglossal duct cyst occurring on the suprasternal region, which is contemplated as an atypical location for TGDCs. This study presents a case report of a female patient, aged 30 years, who presented with a suprasternal swelling and was diagnosed with suprasternal thyroglossal duct cyst (TGDC) through histopathological examination at a teaching hospital. The atypical location of the swelling and difficulty of diagnosis made us report this case.

This case was reported in accordance with the SCARE 2020 criteria [4].

## 2 Case presentation

A 30-year-old female farmer from the rural areas of Ethiopia presented with suprasternal notch swelling since birth, with a recent enlargement in size in the last two years. She had mild discomfort and difficulty of breathing. There were no symptoms of hyperthyroidism or hypothyroidism. No relevant past medical or surgical history was gained from the patient. There was no reported family history of similar illness.

**Fig. 1:**
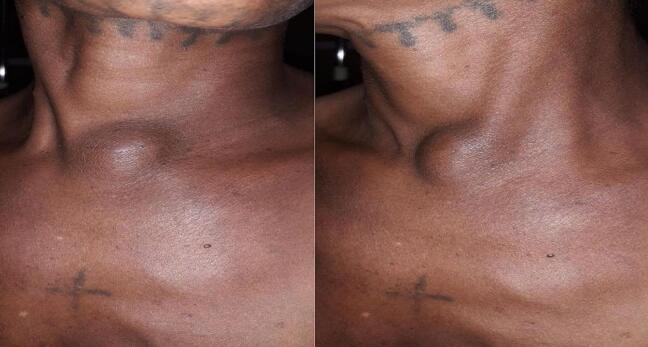
Preoperative photos showing the suprasternal swelling.

On examination, she had a round non-tender midline cystic swelling over the suprasternal notch measuring 6 cm in it's largest diameter, which did not move with swallowing or tongue protrusion (Fig. 1). No gross abnormalities were detected on other systems. On investigation, complete blood count was normal, and neck ultrasonography showed a 5.2 × 4.8 cm well-defined cystic lesion at the suprasternal region inferior to the left thyroid lobe and with internal echogenic foci and high-level echo-debris with peripheral color flow and a multinodular goiter in its normal position. The fine needle aspiration cytology (FNAC) report was a benign cyst, likely a thyroid cyst. Thyroid function test results were in the normal range. Considering the presentation of the patient, the radiological examination results and the location of the cyst, surgical management was decided, and resection of the cyst was done under general anesthesia.

**Fig. 2:**
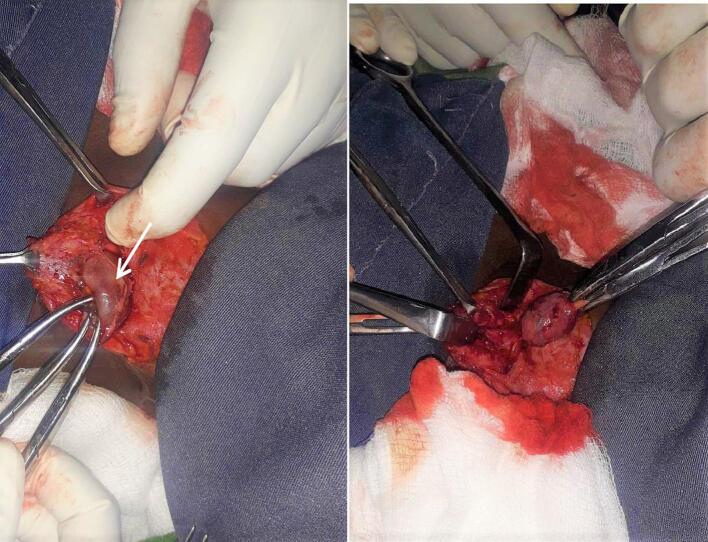
Intraoperative photos (white arrow shows the cystic swelling at the suprasternal area).

**Fig. 3:**
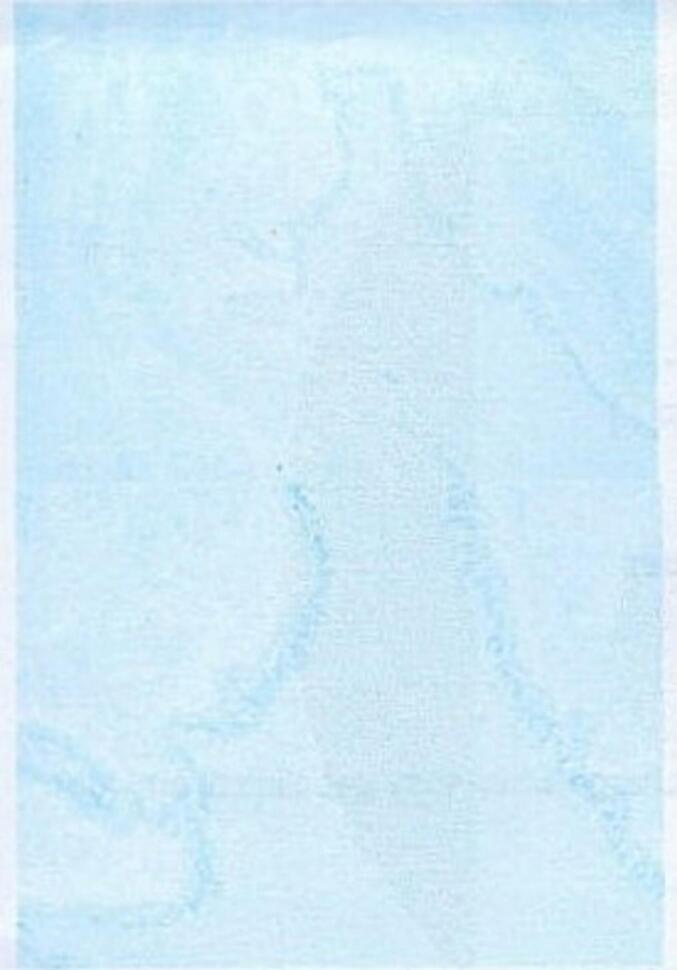
Microscopic slides of the specimen consistent with thyroglossal duct cyst.

Intraoperatively, there was a large cystic mass running from the inferior surface of the thyroid isthmus to the sternum level, and no tract was pointed out (Fig. 2). Therefore, only complete excision of the cyst was done and the specimen was sent for histopathology. The histopathologic slides showed a cyst wall lined by stratified ciliated columnar epithelium in a fibrous stroma with mononuclear inflammatory infiltrates featuring throglossal duct cyst, and no features of malignancy were seen (Fig. 3). The patient had an uneventful postoperative period and was discharged with oral antibiotics on the third postoperative day.

## 3 Discussion

A thyroglossal duct cyst refers to the appearance of a cyst like swelling when residual thyroid tissue remains from the root of the tongue (foramen cecum of tongue) to the anterior sub-hyoid bone during the descent of the thyroid gland [5]. Thyroglossal duct cysts are 85 % of the time found in juxtaposition to the hyoid bone, but can be found anywhere along the descent path of the thyroid gland [6]. TGDC develops in four general locations, the intralingual (2.1 %), suprahyoid (24.1 %), thyrohyoid (60.9 %), and suprasternal areas (12.9 %) [2]. However, there are only two reported cases of a Thyroglossal duct cyst in the suprasternal region, as evidenced in the present case.

Thyroglossal duct cysts are seen in children under 6 years of age in 76 % of cases, although they have also been identified in the fetus in utero [2]. Like in our patient, noticing Thyroglossal duct cyst in the late second decade of life or later is a rarity. Most of the time, they are asymptomatic but may occasionally be infected by bacteria in the oral cavity, prompting the patient to seek medical care [6]. However, various symptoms might be there, like dysphagia, airway obstruction, and obstructive sleep apnea depending on the location of the Thyroglossal duct cyst [7]. The cyst moves upward on protruding the tongue, which is virtually pathognomonic of it [2]. In the present case, the patient had difficulty breathing and pain and the swelling didn't move upward with tongue protrusion. In pediatric patients, ultrasonography can be a valuable tool as a diagnostic modality and usually shows a well defined anechoic to hypoechoic structure with posterior through transmission [5].

Cysts in the suprasternal area can easily be mistaken with a non-specific thyroid gland enlargement (Goiter) as it is the most frequent diagnosis [3]; some of the differentials based on the location of the swelling include dermoid and epidermoid cysts, branchial cleft cysts, laryngocele, thymus gland cysts, malformations of the lymphatic system, and lymph nodes which are necrotic [5]. When TGDCs occur in middle age individuals like our patient, the challenges of settling the diagnosis are doubled. The most accurate hints for diagnosing TGDCs are the histological findings, which show walls made up of alternating non-keratinizing stratified squamous epithelium and epithelium of the respiratory tract [8].

Thyroglossal duct cyst is generally treated surgically and the indications include prevention of infection, for cosmesis, and to avoid the development of carcinoma (which is seen in 1 % of cases) [2]. The treatment choice for a TGDC includes the Sistrunk procedure, which is en bloc excision of the cyst and the central hyoid bone in an effort to minimize recurrence [6]. The recurrence rate with sistrunk procedure based on different studies is from 3 % to 4 %. Local cyst excision on the other hand, is associated with an increased risk of recurrence [9]. In the absence of a duct during surgery, only the cystic mass is excised [5]. We didn't find a thyroglossal duct tract during surgery. Thus, excision of the mass was done. Postoperatively, the histological findings suggested a Thyroglossal duct cyst and no features of malignancy were identified and so no further management was undertaken. The patient is currently on follow-up. One of the commonest postoperative complications that can occur when the Sistrunk procedure was not performed is a thyroglossal duct fistula which can be seen in as high as 85 % of patients [3]. The patient had a smooth recovery and had no signs or symptoms of a thyroglossal duct fistula in the first five months of her follow-up period. In this case, the peculiar features were the location of the swelling, age of presentation and that the swelling did not move upward with protrusion of the tongue, which is virtually a pathognomonic sign of a Thyroglossal duct cyst [2], hence making the diagnosis difficult and the case reportable.

## 4 Conclusion

Even though a thyroglossal duct cyst at the suprasternal area is extremely rare, it is still a possibility and should be in the list of surgeon's differentials diagnosis for a suprasternal mass even in middle age patients with midline lower neck swelling.

## Ethical approval

In our institution study of case reports are exempted from ethical approval in a circumstance that the patient give consent or the guarantee.

## Funding

This research did not receive any specific grant from funding agencies in the public, commercial, or not-for-profit sectors.

## CRediT authorship contribution statement

Dereje Gashaw: Conceptualization, Validation, Data Curation, Writing - Original Draft, Writing - Review & Editing.

Yonas Tesfaye: Conceptualization, Data Curation, Writing - Review & Editing.

Tizazu Yigzaw: Data Curation, Writing - Review & Editing.

Mulat Agalu: Data Curation, Writing - Review & Editing.

Melesse Gebeyehu: Data Curation, Writing - Review & Editing.

Amanuel Sisay: Data Curation, Writing - Review & Editing.

## Guarantor

Dereje Gashaw Andargie, Corresponding author.

## Registration of research studies

1. Name of the registry: Researchregistry.com
2. Unique identifying number or registration ID: researchregistry9238

## Informed consent

Written informed consent was obtained from the patient for publication of this case report and accompanying images. A copy of the written consent is available for review by the Editor-in-Chief of this journal on request.

## Declaration of competing interest

None.
